# The* Mc4r* gene is responsible for the development of experimentally induced testicular teratomas

**DOI:** 10.1038/s41598-023-32784-1

**Published:** 2023-05-01

**Authors:** Syunsuke Seki, Kaoru Ohura, Takehiro Miyazaki, Abdullah An Naser, Shuji Takabayashi, Eisei Tsutsumi, Toshinobu Tokumoto

**Affiliations:** 1 Department of Bioscience, Faculty of Science, Shizuoka University, Shizuoka, 422 Japan; 2 Integrated Bioscience Section, Graduate School of Science and Technology, National University Corporation Shizuoka University, Ohya 836, Suruga-Ku, Shizuoka, 422-8529 Japan; 3 Laboratory Animal Facilities & Services, Preeminent Medical Photonics Education & Research Center, Hamamatsu University School of Medicine, 1-20-1, Handayama, Higashi-Ku, Hamamatsu, Shizuoka 431-3192 Japan; 4 Biological Science Course, Department of Science, Graduate School of Integrated Science and Technology, Shizuoka University, 836 Ohya, Suruga-Ku, Shizuoka, 422-8529 Japan; 5 Present Address: Department of Molecular Genetics, Graduate School of Medicine, Kyoto University, Yoshida Konoe, Sakyo, Kyoto, 606-8501 Japan

**Keywords:** Oncogenesis, Testicular cancer

## Abstract

Teratomas in mice, composed of different tissue types, are derived from primordial germ cells in the fetal gonads. Previously, we identified a locus responsible for experimental testicular teratoma (ETT) formation on chromosome 18, referred to as *ett1*. The strongest candidate sequence in the *ett1* locus was found to be a missense mutation in the melanocortin 4 receptor (*Mc4r*)*, Mc4r*^*G25S*^. We established a strain with a point mutation in the *Mc4r* gene in the ETT-nonsusceptible LT strain, called LT- *Mc4r*^*G25S*^, by genome editing. Surprisingly, highly developed ovarian teratomas (OTs), rather than testicular teratomas, appeared in the LT-*Mc4r*^*G25S*^ strain. The results demonstrated that *Mc4r* is also one of the genes responsible for OT formation and suggested that missense mutations in *Mc4r* promote teratoma formation in both sexes. In this study, we performed ETT experiments in different host–graft combinations of the LT-*Mc4r*^*G25S*^ and LT strains. Furthermore, the expression of MC4R in germ cells in the testis was demonstrated. Expression of *Mc4r* in testis was also confirmed by RT-PCR. The results demonstrated that MC4R is expressed in germ cells in the testis and that a point mutation in the *Mc4r* gene is responsible for ETT formation.

## Introduction

A teratoma is a tumor of germ cell origin containing tridermic tissues. Teratoma formation has been reported in mice as well as in humans and birds. Teratomas occur congenitally but can also be induced experimentally. Teratoma formation is also used to verify whether stem cells retain pluripotency. Teratomas are formed when pluripotent cells (embryonic carcinoma cells (EC cells), embryonic stem cells (ES cells), induced pluripotent stem cells (iPS cells)) are transplanted into differentiated tissues^1–4^. Spontaneous testicular teratoma (STT), an inborn teratoma formed in the mouse testis, is a differentiated pluripotent tumor containing tridermic tissues derived from primordial germ cells (PGCs). It has been suggested that 6–8 recessive loci are involved in the formation of STTs in the 129/Sv mouse strain^5^. One of these strains, the 129/Sv-+/*Ter* strain bearing the *Ter* mutation on chromosome 18, is known to have an increased rate of STT formation^6,7^. Noguchi and Noguchi established that the 129/Sv-+/*Ter* strain develops STTs at a rate of almost 100%^8^. The *ter* mutation was identified in 2005 as a nonsense mutation in the *dead end 1* (*Dnd1*) gene, a homolog of the dead end (*dnd*) gene that causes primordial germ cell defects in zebrafish^9,10^.

Furthermore, the formation of experimental testicular teratoma (ETT) can be induced in the 129 subline by implanting the testis of a 12.5-day embryo (E12.5) into an adult testis^11^. Stevens performed an experiment in which fetal testes from 129/Sv-C P Sl mice that carried the Steel (*Sl*) mutation in the *kit* gene were implanted into adult testes. Mice with a homozygous *Sl*/*Sl* genotype develop germ cell defects. However, the wild-type and *Sl*/ + genotypes had a teratoma formation rate of 70–80%, while mice with the *Sl*/*Sl* genotype almost never formed teratomas^12^. This experiment showed that the teratoma was of germline origin. The rate of ETT formation also depends on the age of the transplanted testes. It was reported that 82% of 12.5-day fetal testes transplanted into adult testes formed ETTs, while the rate of ETT formation decreased to 8% when E13.5 fetal testes were transplanted^11^. Noguchi et al*.* transplanted fetal testes from a *Ter* congenic line (B6-+/Ter) and found that ETTs were not formed^13^. These results indicate that other genes are responsible for ETT formation^14,15^. In addition, when fetal testes from 12.5-day embryos of 129/Sv-+*/Ter* wild-type (+/+) mice were transplanted, ETTs formed 80–90% of the time, even though they did not carry the *Ter* mutation. However, transfer of fetal testes from B6 or LTXBJ(LT)^16^ rather than the 129 strain does not result in the formation of ETTs. The fact that STTs and ETTs are not formed in the *Ter/Ter* and + */Ter* congenic lines and that ETTs are formed when a fetal testis from the wild-type *Ter* 129 is transplanted into an adult testis indicates that there are other genes in the genetic background of line 129 that are responsible for ETT formation. We have focused on identifying the causative genes and elucidating the mechanism of teratoma formation in ETT.

The F2 generation was obtained by crossing the F1 [LTXBJ × 129/Sv-*Ter*^+/+^] x F1 [LTXBJ × 129/Sv-*Ter*^+/+^] generations of the teratoma-forming line 129 and the nonteratoma-forming line LT to identify the region in which the gene responsible for experimental testicular teratoma formation resides. Fetal testes were transplanted into F1 adult testes for teratoma formation assays, followed by simple sequence length polymorphism (SSLP) genotyping and linkage analysis using microsatellite markers on chromosomes 1 through 19. Three regions containing candidate causative genes for experimental testicular teratoma, named experimental testicular teratoma 1, 2, 3 *(ett1, ett2,* and *ett3*), were identified on chromosomes 18, 3, and 7, respectively^17,18^. Exome sequencing analysis was performed to search for mutations in these three regions, and 129-lineage-specific SNPs were identified, including one in *Mc4r* in the *ett1* region^18^. In a previous study, we established a gene-edited strain, LT-*Mc4r*^*G25S*^, by using an electroporation-based gene transfer method (TAKE)^20^. Surprisingly, highly developed ovarian teratomas (OTs) formed in the female mice of this strain^21^. This result demonstrated that *Mc4r* is one of the genes responsible for ovarian teratoma formation.

*Mc4r* is involved in energy intake and energy expenditure; thus, the gene knockout strain shows an obese phenotype^22,23^. As already mentioned in a previous paper, we hypothesized that instead of melanocyte-stimulating hormone (MSH), an authentic ligand for MC, agouti and agouti-related protein (AGRP) is thought to be a ligand for MC4R expressed in mouse germ cells^21^. One piece of supportive knowledge is that agouti gene mutation causes a tenfold lower rate of testicular teratoma formation^24^. Ay is a dominant mutation in the agouti gene and causes ectopic expression of agouti protein, now it is referred to agouti-signaling protein-1 (ASIP1), and the inhibition of MC1R and MC4R chronic signal transduction systems^25–27^. ASIP1 is an antagonist for MC4R and high expression of ASIP1 in agouti-mice (Ay/a) resulted in obese phenotype as *Mc4r*^*-/-*^ mice^28^. The increased expression of *Mc4r* in agouti-mice was demonstrated and suggested that it was the compensatory mechanism for prevention of ASIP1-mediated inhibition of the MC4R-signaling in the obese agouti-mice. AGRP was found as antagonist for MC3R and MC4R^29^. AGRP was subsequently shown to act as an inverse agonist against for MC4R^30^. AGRP has also been shown to act as a biased agonist for MC4R^31^. Interestingly, expression of *asip1* and *agrp* detected from fetal testis and its expression continue to adult^32^. It is possible that these ligands regulate the germ cell survival, division or apoptosis through MC4R-signaling.

In this study, we investigated whether *Mc4r*^*G25S*^ is responsible for ETT formation. Transplantation of fetal testes from *Mc4r*^*G25S*^ mice into adult testes in *Mc4r*^*G25S*^ mice resulted in a high rate of ETT formation. These results demonstrate that *Mc4r*^*G25S*^ is responsible for ETT formation.

## Results

**Figure 1:**
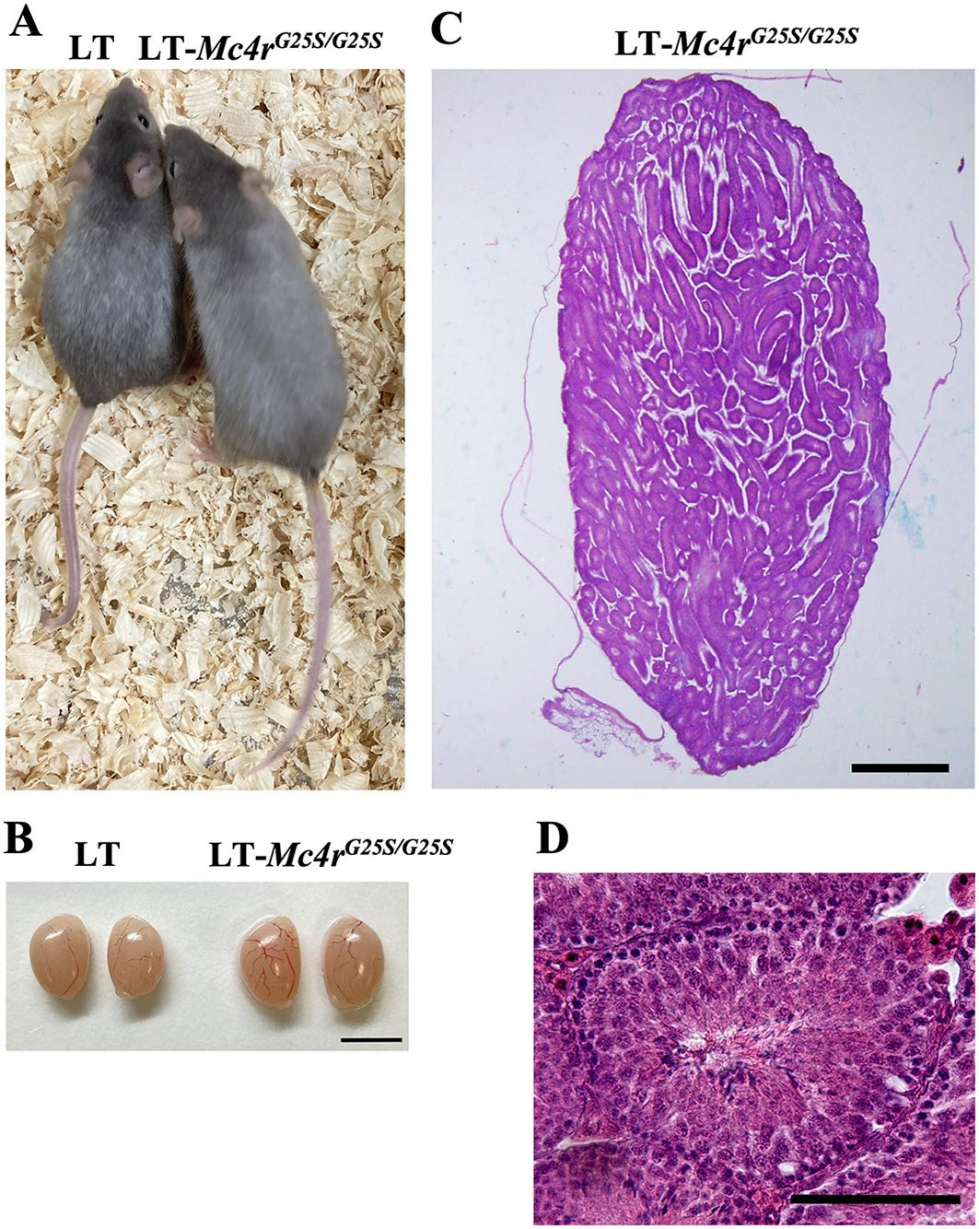
STT was not found in LT-*Mc4r*^*G25S/G25S*^ male mice. (**A**) Photographs of LT and LT-*Mc4r*^*G25S/G25S*^ male mice. (**B**) Morphology of the testes of LT-*Mc4r*^*G25S/G25S*^ and LT. Scale bar = 5 mm. (**C**) A section of testis from a LT-*Mc4r*^*G25S/G25S*^ mouse. Scale bar = 500 μm. (**D**) A section of seminiferous tubule. Scale bar = 50 μm.

Strains homozygous for a knock-in mutation substituting G in LT to A in 129 to produce *Mc4r*^*G25S*^ were established by the TAKE (Technique for Animal Knockout system by Electroporation) method^20^ as previously described^21^ (Fig. 1A). The LT*- Mc4r*^*G25S*^ strain females showed OT formation. In contrast, no obvious teratoma formation was observed in the males, with no incidence of STT found in testis sections among hundreds of mice during breeding (Fig. 1B,C). Thus, the SNP *Mc4r*^*G25S*^ is not sufficient for STT formation in the LT genetic background. We conducted transplantation experiments to determine whether the SNP *Mc4r*^*G25S*^ is responsible for experimental testicular teratoma (ETT) formation.

**Figure 2:**
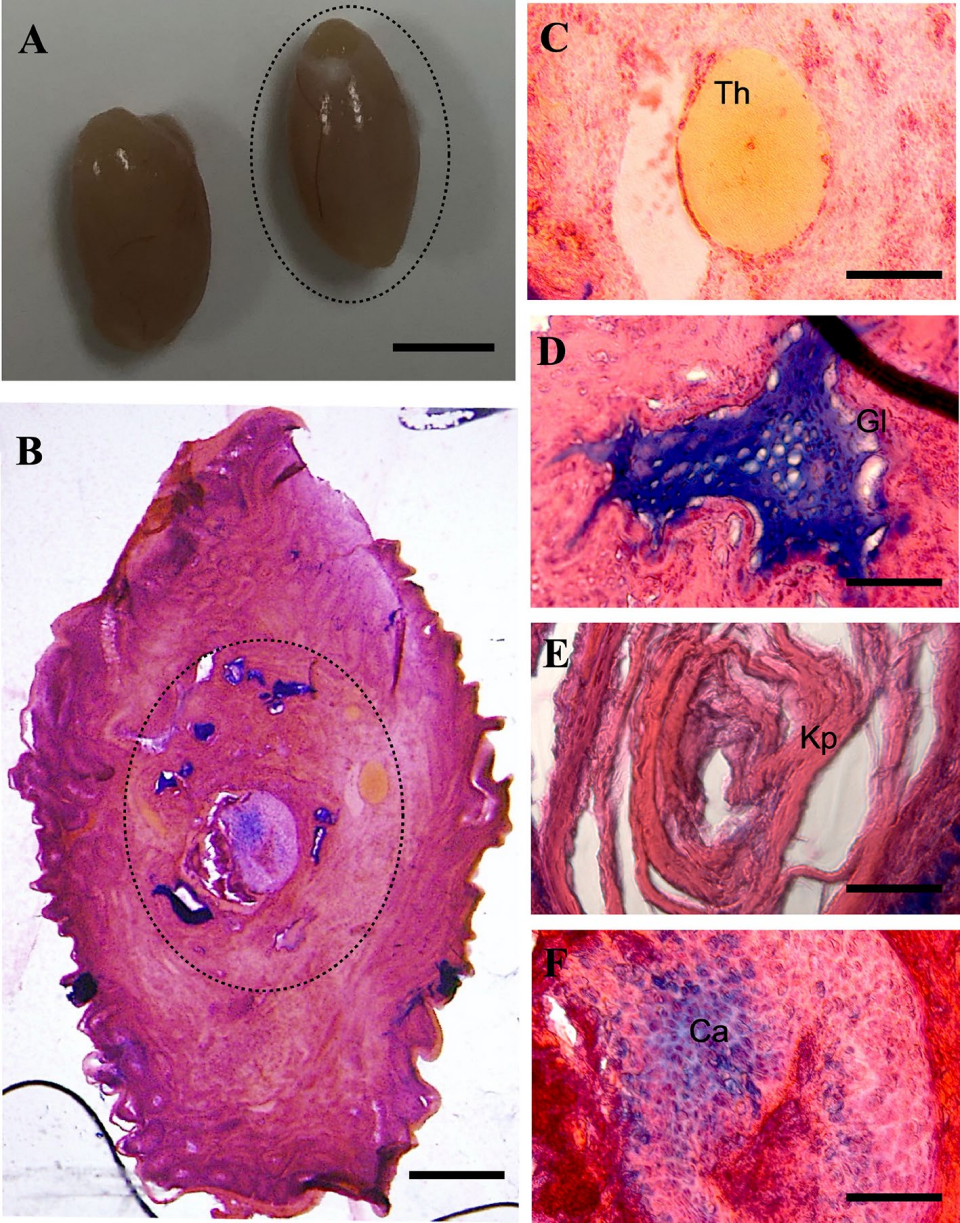
ETT formation by transplantation of fetal testis from LT-*Mc4r*^*G25S/G25S*^ into LT-*Mc4r*^*G25S/G25S*^. (**A**) Morphology of the ETT-forming testes. The right testis was transplanted and sectioned. Scale bar = 5 mm. (**B**) A testicular teratoma was found in a testis section (the teratoma area developed from transplanted fetal testis is indicated by the dotted circle). Scale bar = 500 μm. (**B**–**E**) Images of tissue-like structures found in a teratoma: (**C**) thyroid follicular cell (Th); (**D**) glandular cell (Gl); (**E**) keratin pearl (Kp); (**F**) cartilage tissue (Ca). Scale bars = 50 μm.

First, we transplanted LT-*Mc4r*^*G25S/G25S*^ fetal testes as donors into LT-*Mc4r*^*G25S/G25S*^ adult testes as hosts (Fig. 2). In the ETT of one specimen, various types of tissues from all three germ layers, such as thyroid follicular cells, glandular cells (endodermal), keratin pearls (ectodermal), and cartilage tissue (mesodermal), were observed (Fig. 2B–F). Although one specimen (shown in Fig. 2) produced a teratoma composed of tissues that could be identified as derived from all three germ layers, teratomas with tissues of only two or one germ layers were found in the other specimens (Supplementary Fig. S1).

**Figure 3:**
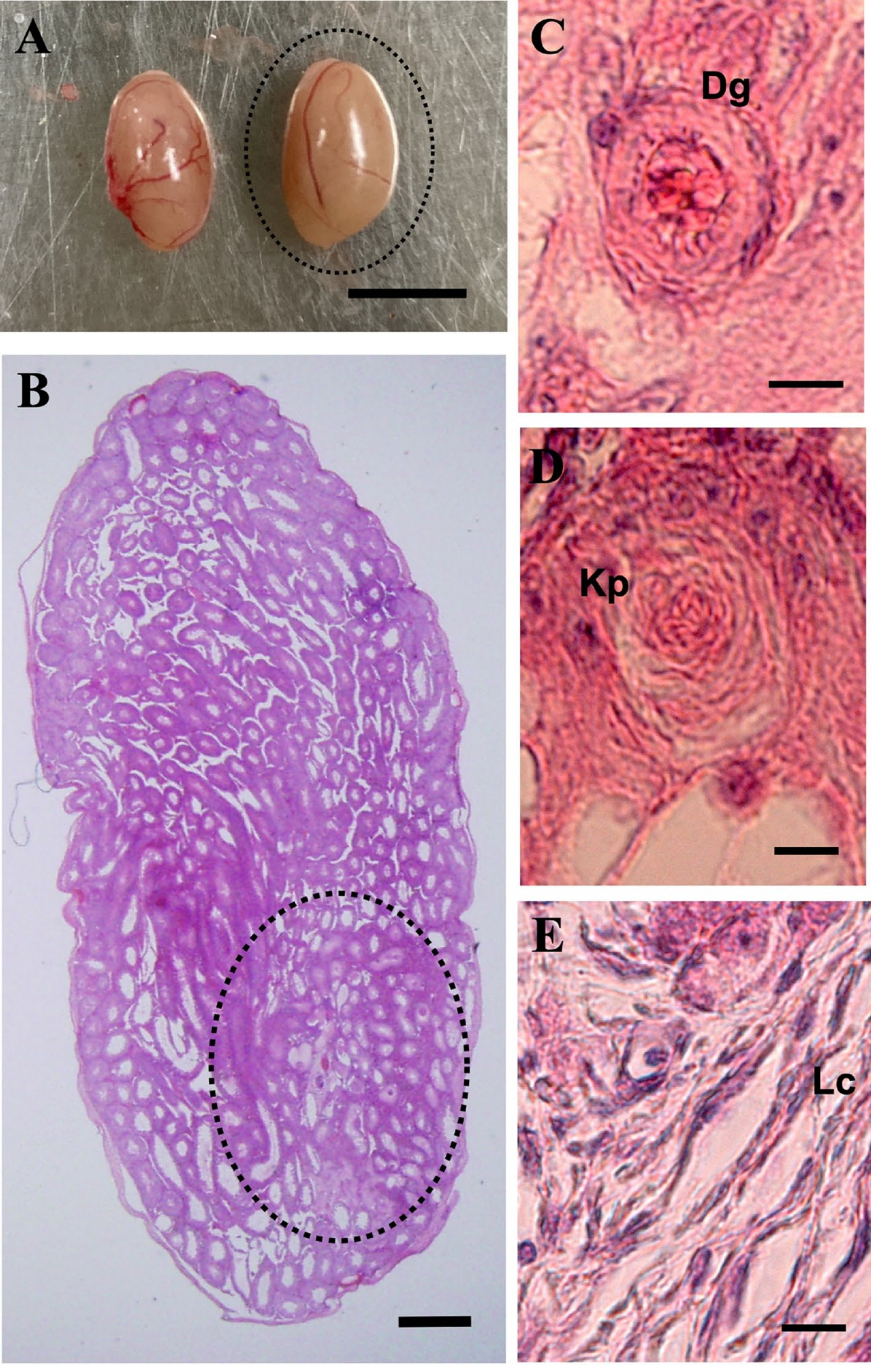
ETT formation by transplantation of fetal testis from LT-*Mc4r*^*G25S/G25S*^ into LT. (**A**) Morphology of the ETT-formed testes. The right testis was transplanted and sectioned. Scale bar = 5 mm. (**B**) A developed testicular teratoma was found in the section of the testis (the teratoma area developed from transplanted fetal testis is indicated by dotted circle). Scale bar = 500 μm. (**B**–**E**) Photographs of tissue-like structures found in a teratoma: (**C**) digestive gland (Dg); (**D**) keratin pearl (Kp); (**E**) loose connective tissue (Lc). Scale bars = 10 μm.

ETTs were also found in another donor–host combination, LT-*Mc4 *^*G25S/G25S*^ fetal testis as a donor into LT adult testis as a host (Fig. 3). In the ETT of one specimen, various types of tissues derived from the three germ layers, such as digestive gland (endodermal); keratin pearls (ectodermal); and loose connective tissue (mesodermal), were observed (Fig. 3).

Although one specimen indicated in Fig. 3 produced a teratoma composed of tissues that could be identified as derived from three germ layers, teratoma with tissues derived from only two or one germ layers were found in other specimens (Supplementary Fig. S2).

The incidence of teratoma in each genotype is summarized in Table 1. In total, 26 *Mc4r*^*G25S*^ fetal testes were transplanted into adult *Mc4r*^*G25S*^ testes, and ETT formation was observed in 6 offspring (23.1%). Similarly, 11 LT-*Mc4r*^*G25S*^ fetal testes were transplanted into LT adult testes, and ETT formation was observed in 3 offspring (27.2%). When LT fetal testes were transplanted into LT-*Mc4r*^*G25S*^ adult testes (10 cases), no ETT formation was observed (0%), nor were ETTs observed after transplantation of LT fetal testes into LT adult testes^17^.

**Table 1:** Incidence of ETT formation in different combinations of grafts and hosts.

graft/host	% of ETTs*	(ETT formation/graft)
LT*-Mc4r*^*G25S*^/LT*-Mc4r*^*G25S*^	23.1	(6/26)
LT-*Mc4r*^*G25S*^/LT	27.2	(3/11)
LT/LT-*Mc4r*^*G25S*^	0	(0/10)

*The male genital ridges of E12.5 embryos were grafted into a host testis using the method described in the Materials and Methods. Graft sites were removed over 8 weeks after surgery and subjected to histological inspection. Grafts carrying teratomas are defined as ETTs.

The incidence of ETTs was approximately the same for grafts of fetal testes of LT-*Mc4r*^*G25S/G25S*^ into LT-*Mc4r*^*G25S/G25S*^ and LT testes. These results suggested that the stimulus for ETT development was derived from LT-*Mc4*^*G25S/G25S*^ fetal testes.

**Figure 4:**
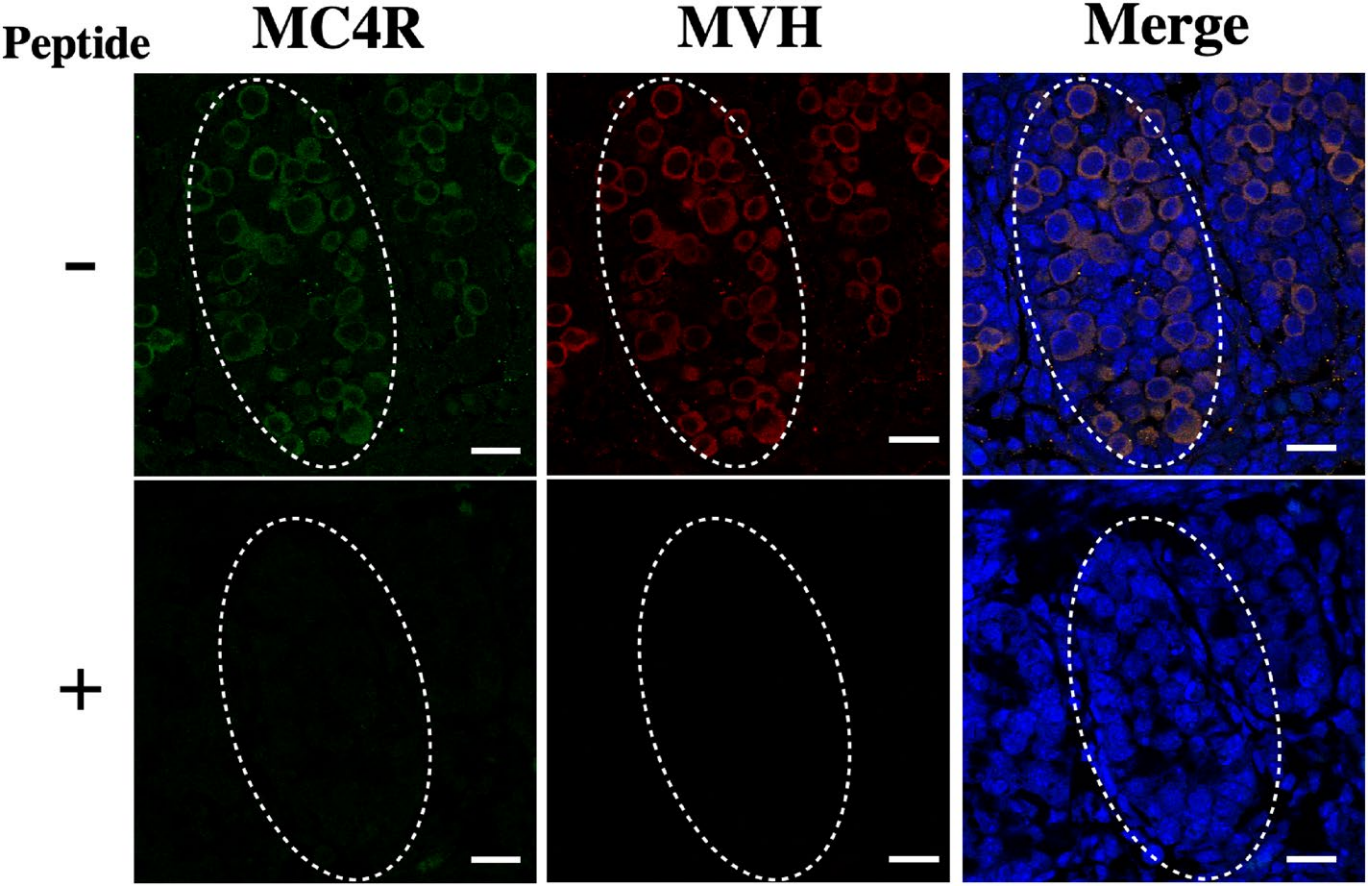
MC4R is expressed in germ cells in the testis. Immunofluorescence staining of sections of fetal testis from the LT strain. Sections were double stained with anti-rabbit MC4R polyclonal antibodies (α-MC4R), anti-mouse MVH polyclonal antibodies (α-MVH) and DAPI. Merged images of both antibodies and DAPI are also shown (Merge). Control staining with peptide-blocked antibodies is shown below each photograph. Forming seminiferous tubules are indicated by dotted circles. Scale bars = 100 μm.

It was demonstrated by immunofluorescence analysis that MC4R was expressed in germ cells in the fetal testis of LT (Fig. 4).

**Figure 5:**
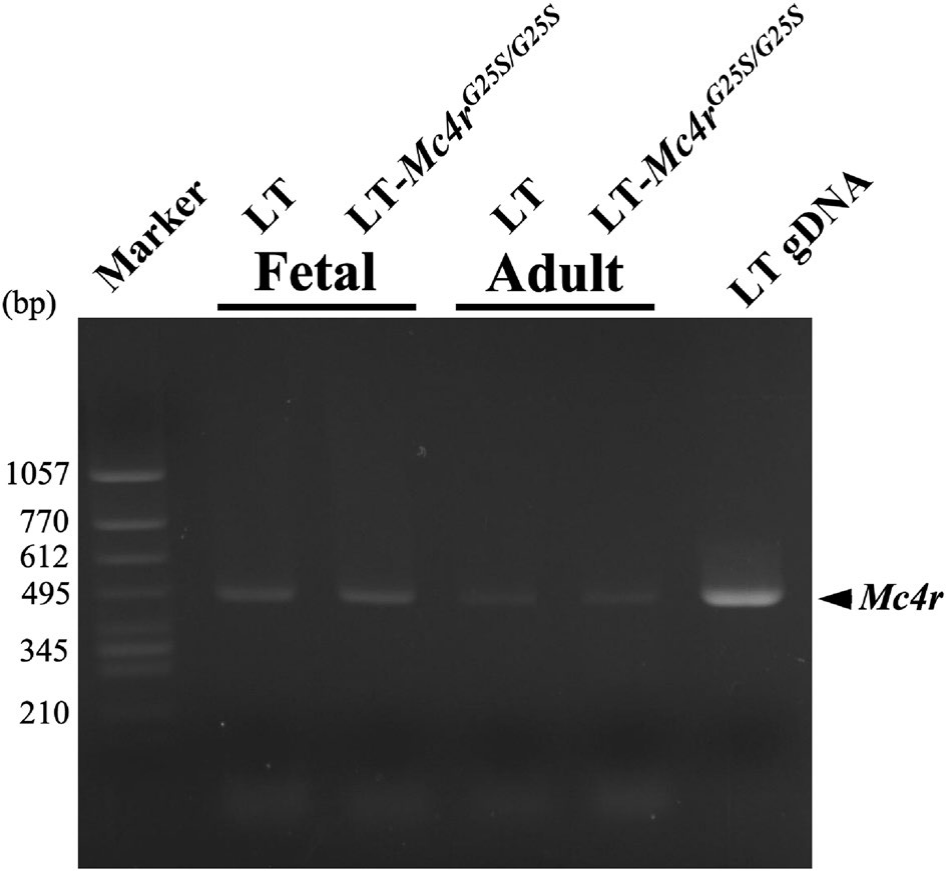
Expression of *Mc4r* in the fetal and adult testis. RT-PCR was performed with total RNAs extracted from fetal and adult testis from the LT and LT-*Mc4r*^*G25S/G25S*^ strains. The amplified band of *Mc4r* is indicated by arrowhead. PCR amplification is also conducted by using genomic DNA from LT strain as template (gDNA). Genomic DNA was isolated from the ear of LT mouse. Full-length gel images for this figure in different capture setting are indicated in Supplemental figures.

The signals representing MC4R and a germ cell-specific protein, mouse vasa-homolog (MVH), overlapped well. In a previous study, we showed that MC4R proteins are expressed in oocytes^21^. We also examined expression of *Mc4r* in testis by RT-PCR. The results indicated that *Mc4r* was expressed in both fetal and adult testis of LT and LT-*Mc4r*^*G25S/G25S*^ (Fig. 5). These results demonstrated that mouse MC4R is expressed in germ cells.

## Discussion

In this study, we identified *Mc4r* as the *ett1* gene responsible for ETT formation. We mapped the *ett1* region, which is a candidate region for genes involved in ETT formation, and found that among the 8 genes existing in the *ett1* region, there is a SNP in *Mc4r* that causes an amino acid change between the 129 strain and the LT strains ^18^. Thus, the single amino acid change in *Mc4r* is proposed to be a candidate for *ett1*. Then, to validate *Mc4r* as the *ett1* gene, a knock-in strain, LT-*Mc4r*^*G25S/G25S*^, in which this single base substitution was introduced into the LT strain, was created by genome editing. The original LT strain was established as a strain susceptible to OTs in which testicular teratomas (STTs and ETTs) do not develop at all. Surprisingly, we found that a knock-in strain, LT-*Mc4r*^*G25S/G25S*^*,* developed OTs at an extremely high rate^21^. The results suggested that *Mc4r* is a gene responsible for OT formation.

The suggested role of MC4R in germ cells is in the antiapoptotic signaling pathway. As indicated in this study, MC4R is expressed in germ cells in fetal testes. Expression of *Mc4r* is also demonstrated by RT-PCR. Furthermore, we already reported of the detection of *Mc4r* expression by qPCR both in fetal and adult testis in 129 and LT strains^18^. It is hypothesized that the modification of the antiapoptotic pathway of testicular germ cells enhances the transdifferentiation of germ cells into somatic cells or increases the rate of parthenogenesis and causes testicular teratoma formation. Regarding the role of DND1 in suppressing the apoptosis of germ cells^33^ or maintaining germ cell fate by suppressing the transdifferentiation of germ cells into somatic cells^34^, the MC4R-mediated signal cascade is involved in germ cell survival, apoptosis and control of the transdifferentiation of germ cells into somatic cells.

These results strongly suggest that *Mc4r*, an *ett1* candidate gene, may be one of the causative genes of STT formation.

In our ETT formation experiments, tridermic teratomas rarely developed. In addition, highly developed teratomas that could be observed externally were not formed. Because the genetic background of this transplantation experiment did not include the *Ter* gene mutation, it is suggested that *Ter* gene mutation is required for the development of highly proliferative and differentiated tridermic teratomas. We also identified two additional ETT-related loci, *ett2* and *ett3*, on chromosomes 3 and 7, respectively. It should be clarified whether and how these loci are involved in the development of ETT.

By this study it was suggested that *Mc4r* is involved in the development of experimental testicular teratoma in mice. It has become clear that *Mc4r*, whose function in the central nervous system has been attracting attention, is also expressed in germ cells and is involved in their control. Teratomas also develop in human testes and ovaries^35^. The *Ter* gene mutation (non-sense mutation of *Dnd1*) discovered in mouse teratoma studies have been investigated as a cause of human cancers and have been shown to have inhibitory effects on several cancers such as liver cancer and tongue cancer^36–38^. Large-scale data analysis of MC4R also points out that a SNIP close to MC4R gene was related to endometrial cancer and breast cancer^39,40^. Thus, the relation between MC4R and human gonadal cancer should be analyzed as a possible causative. Data from cBioPortal, which has been opened as a platform for analyzing gene mutations in actual human tumor samples, shows that DND1 has a mutation rate of 0.84% in testicular germ cell tumors (TGCTs)^41^, while MC4R has a mutation rate of 2.01% (cbioportal.org, last accessed on 2 Mar 2023)^42,43^. All alteration (mutation, deletion or amplification) of DND1 in TGCTs samples was mutations, whereas deep deletion was all the case of MC4R. These patterns of alteration in DND1 and MC4R were specific for TGCTs among patterns of alteration found in various cancers. These specific mutational patterns may imply specific functions of DND1 and MC4R in the gonad. A detailed analysis of the relationship between MC4R and human TGCTs should be carried out.

## Methods

### Mice

All animals were housed under temperature-controlled conditions and had free access to food and water. All animal procedures were performed per the protocols approved by the Institutional Animal Care and Use Committee of Shizuoka University (2021A-9, 2022A-5). All experiments were performed in accordance with the guidelines of the committee. The study was carried out in compliance with the ARRIVE guidelines^44^.

The 129/Sv-*Ter* and LTXBJ mice^16^, generous gifts from Dr. Leroy Stevens (The Jackson Laboratory, Bar Harbor, ME, USA), were bred in the animal facilities at Shizuoka University. Males of the LT strain undergo normal spermatogenesis and do not form STTs or ETTs^13^. The genome-edited LT- *Mc4r*^*G25S/G25S*^ strain was established by the TAKE method, as previously described^21^. The F1 offspring obtained were genotyped by sequence analysis, and offspring of the same genotypes were submitted to sibling mating.

### Transplantation of fetal testis into adult testis

To address ETT formation ability, transplantation of fetal testis into adult testis was performed as previously described^17^ except that the transplanted testis were excised 2–3 months after transplantation to analyze ETT formation in this study.

### Observation of testicular teratomas

For each mouse, the testis morphology to three months after transplantation was observed and photographed after dissection. Whether the teratoma developed on the left side, right side, or both sides was recorded.

### Histological analysis

Tissues were dissected and fixed in Bouin's solution, embedded in paraffin, and serially sectioned at 6-μm thickness. Deparaffinized sections were stained with hematoxylin, eosin and alcian blue (H-E-A) and screened for the presence of ETTs under a light microscope.

### Immunofluorescence observation

The testes were fixed with 4% PFA, dehydrated, cleared, and embedded in paraffin to prepare 5 μm-thick sections. After deparaffinization, the slides were autoclaved with 10 mM sodium citrate buffer (pH 6.0) for 10 min at 120 ℃ for antigen retrieval. Slides were washed 3 times with TBST, and then the tissue was surrounded with a Pap Pen liquid blocker (DAIDO SANGYO CO., LTD, Tokyo Japan). Sections were blocked with 3% BSA/TBST for 1 h at room temperature. The primary antibodies anti-MC4R (1:80, Cayman Chemical) and anti-DDX4/MVH (1:50, Abcam) were diluted in 3% BSA/TBST and incubated with the tissue sections overnight at 4 °C. Sections were washed 3 times with TBST for 10 min each. Then, the secondary antibodies, anti-rabbit IgG (H + L), F(ab')2 fragment (Alexa Fluor 488 conjugate) (1:500, Cell Signaling), anti-mouse IgG (H + L), and F(ab')2 fragment (Alexa Fluor 555 conjugate) (1:500, Cell Signaling), diluted in 3% BSA/TBST, were applied at room temperature for 1 h. After 3 washes with TBST for 10 min each, sections were mounted in a DAPI-containing water-soluble mountant (ProLong). A Carl Zeiss confocal laser scanning microscope (LSM700) was used for fluorescence observation.

### Reverse transcription-polymerase chain reaction (RT-PCR)

Total RNA was isolated from fetal or adult testis using pre-made reagents for RNA extraction (ISOGEN, Nippon Gene, Tokyo, Japan) in accordance with the manufacturer’s protocol. RT-PCR was carried out using Ready-to-Go RT-PCR Beads (Amersham Biosciences) in accordance with the manufacturer’s instructions. The gene-specific primers used for *Mc4r* were as follows: (sense 5′-AAAGGAGGCAAAAACAGGCG-3′ and antisense 5′-CAGGACCAAATGTGTCCGTT-3′). PCR conditions involved incubation for 30 s at 95 °C, 30 s at 67 °C and 60 s at 72 °C for a total of 40 cycles.

## Supplementary Information

Supplementary Information.

## Data Availability

The datasets generated and/or analyzed during the current study are available from the corresponding author on reasonable request.
