# Supplementary material for: The Mc4r gene is responsible for the development of experimentally induced testicular teratomas
Source: Sci Rep. 2023 May 1;13:6756. doi: 10.1038/s41598-023-32784-1 (PMC10151343; doi:10.1038/s41598-023-32784-1)

Supplementary Information for

The *Mc4r* gene is responsible for the development of experimentally induced testicular teratomas

Syunsuke Seki[1]*, Kaoru Ohura[1]*, Takehiro Miyazaki[2+], Abdullah An Naser[1], Shuji Takabayashi[3], Eisei Tsutsumi[4], Toshinobu Tokumoto[1,2,4]

[1]Department of Bioscience, Faculty of Science, Shizuoka University, Shizuoka 422, Japan.
[2]Integrated Bioscience Section, Graduate School of Science and Technology, National University Corporation Shizuoka University, Ohya 836, Suruga-ku, Shizuoka 422-8529, Japan.
[3]Laboratory Animal Facilities & Services, Preeminent Medical Photonics Education & Research Center, Hamamatsu University School of Medicine
1-20-1, Handayama, Higashi-ku, Hamamatsu, Shizuoka 431-3192, Japan
[4]Biological Science Course, Department of Science, Graduate School of Integrated Science and Technology, Shizuoka University, 836 Ohya, Suruga-ku, Shizuoka, 422-8529 Japan

+Present address: Department of Molecular Genetics, Graduate School of Medicine, Kyoto University, Yoshida Konoe, Sakyo, Kyoto 606-8501, Japan
*These two authors contributed equally to this study.

Correspondence tokumoto.toshinobu@shizuoka.ac.jp

Figure caption of Supplementary Figures

Supplementary Figure. S1

ETT formation by transplantation of foetal testis between LT- *MC4R*$^{G25S/G25S}$ and LT-*MC4R*$^{G25S/G25S}$ strain. Left side; Photographs of tissue sections from five specimens that formed ETT by transplantation (individual number of mouse and side of testis are indicated on left side). Teratoma developed area from transplanted fetal testis is indicated by dotted circle. Scale bar = 1 mm. Right side; Enlarged photographs of tissue-like structures found in a teratoma: neuroepithelium (Ne)
; thyroid follicular cell (Th); neuroglia cell (Ng); adipose tissue (Ad); blood (Bl); blood vessel (Bv). Scale bars = 100 μm.

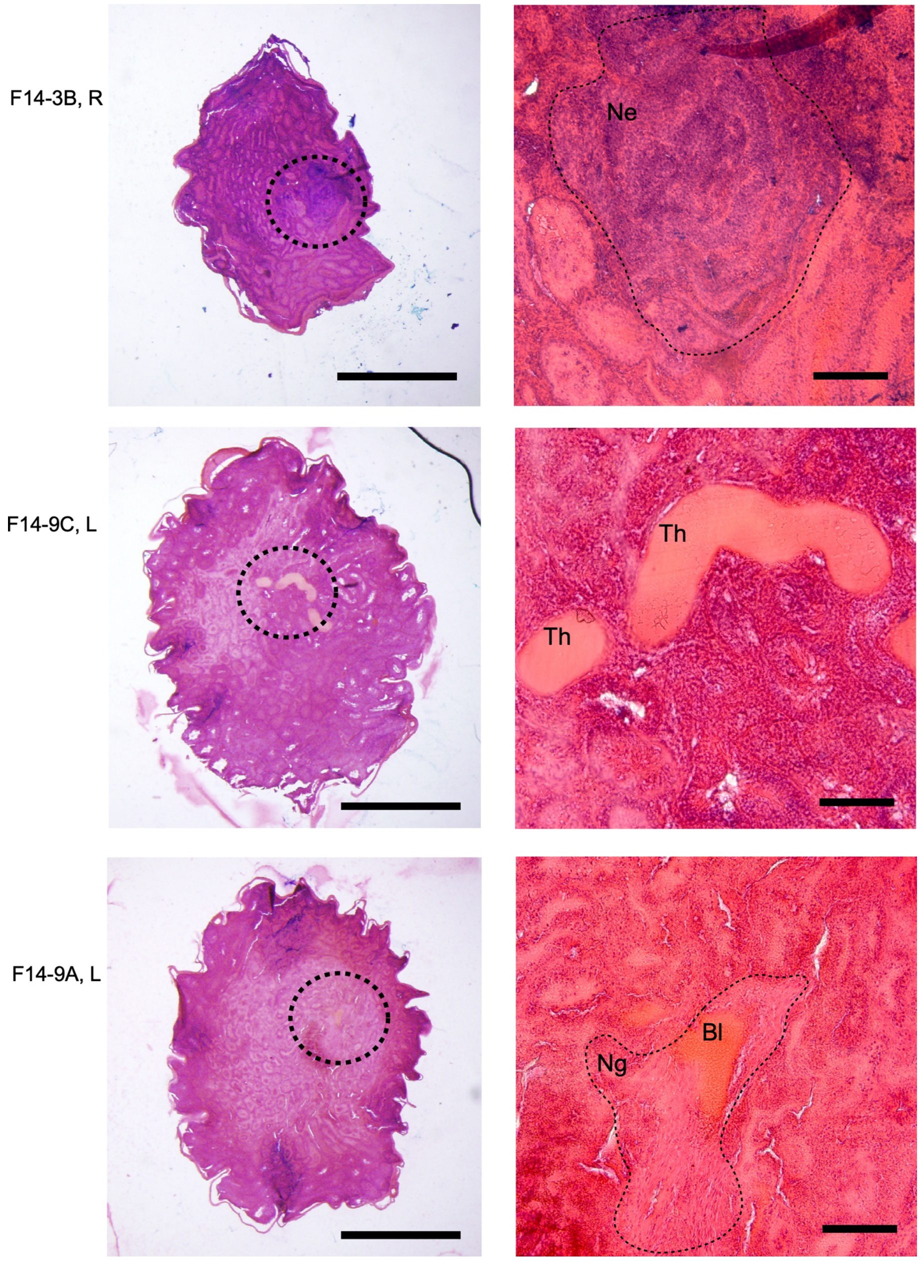

F14-3F, R

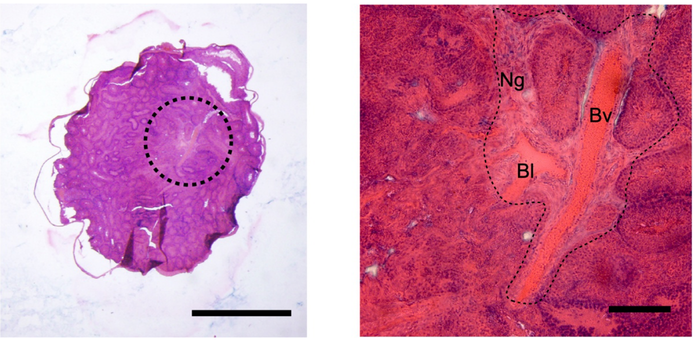

F14-1B, L

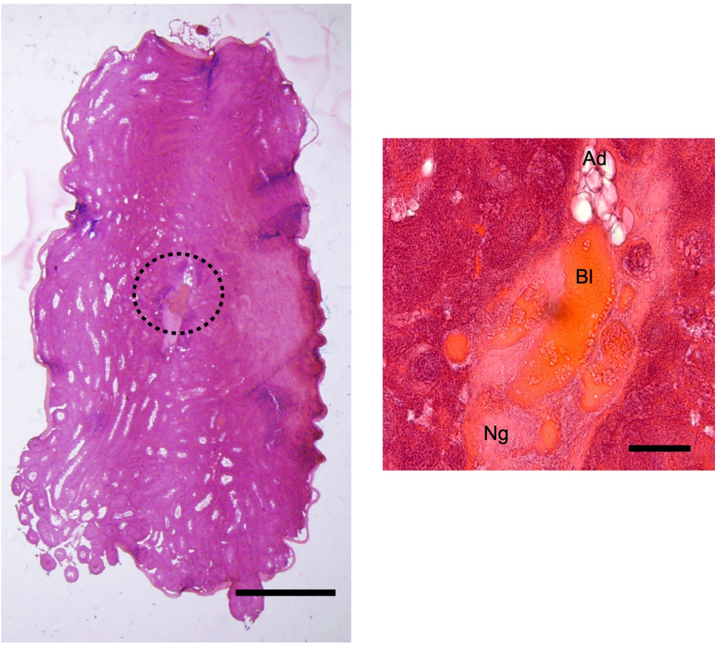

Supplementary Figure. S2

ETT formation by transplantation of foetal testis between LT- *MC4R*$^{G25S/G25S}$ and LT strain. Left side; Photographs of tissue sections from two specimens that formed ETT by transplantation (individual number of mouse and side of testis are indicated on left side). Teratoma developed area from transplanted fetal testis is indicated by dotted circle. Scale bar = 1 mm. Right side; Enlarged photographs of tissue-like structures found in a teratoma: neuroglia cell (Ng), cystic fluid (Cf), keratinized cyst with keratinous substances (Kcy); thyroid follicular cell (Th); blood(Bl). Scale bars = 100 μm.

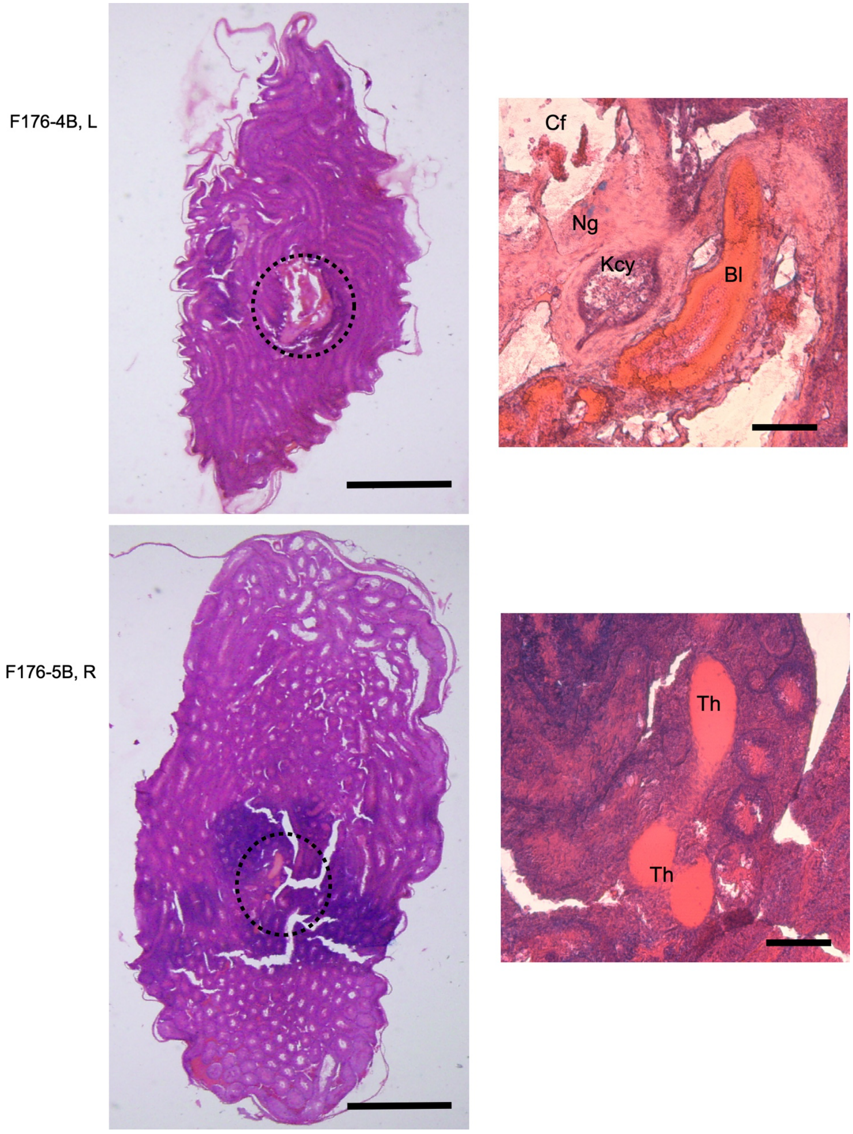

Full-length gel images for Figure 5.

Gel images were captured in several different setting. The most upper picture was indicated in Figure. 5.

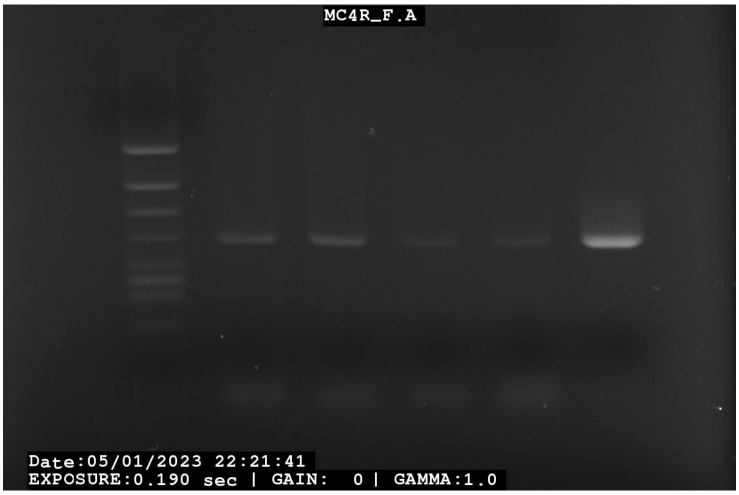

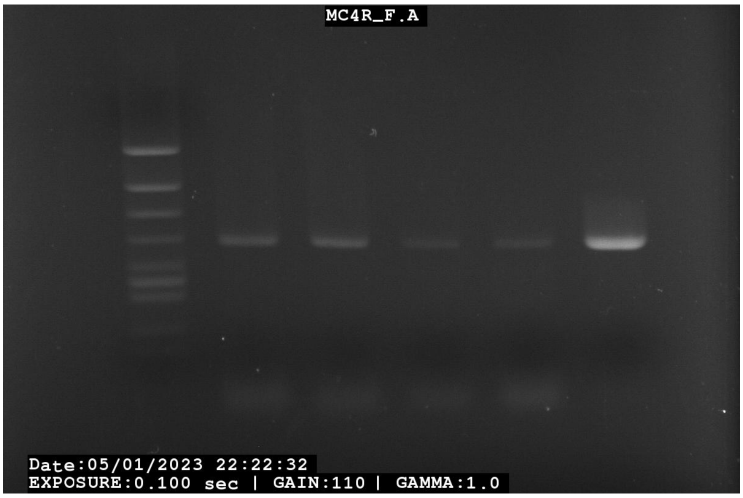

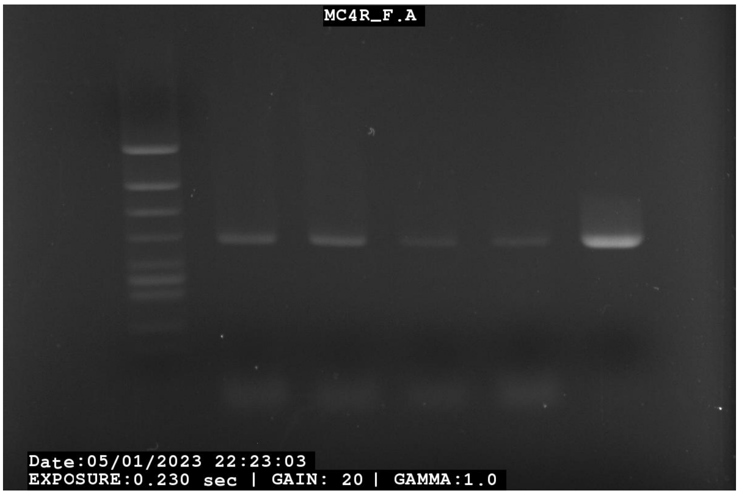

Supplement: Supplementary file 1 — Supplementary Information. [file 41598_2023_32784_MOESM1_ESM.pdf]
